# Practical Notes on Diagnosis and Treatment

**Published:** 1910-04-02

**Authors:** 


### Practical Notes on Diagnosis and Treatment.

**Abdominal Tuberculosis.**

When tuberculosis in children appears clinically to be primary in the abdomen, the results of autopsies show that in over half the cases the infection is limited to the belly.

**Brachial Neuritis.**

The affected limb must be given complete and absolute rest, by means of a sling or by bandaging the arm to the side. Counter-irritation is a second factor of importance, beginning with mustard leaves and, if necessary, pressing on to the thermo cautery, to be applied to the tender points. Of internal remedies I have obtained the most satisfactory results with a combination of sodium salicylate with potassium iodide and bromide.—*Dr. Aldren Turner.*

**Vascular Changes in the Aged.**

Cramps in the legs are common in old people, especially at night; limb weariness is experienced after slight effort, local neuralgias are frequent, and often cause considerable suffering. These are mainly due to partial obstruction of the circulation, causing anæmia of muscles, which renders their nutritional changes insufficient in quantity and rapidity to maintain power. It is a curious clinical fact that in old people, even after considerable atheromatous change in their vessels, the muscular coats of the arterioles to a great extent escape or remain susceptible to vasomotor contraction.—*Dr. R. Douglas Powell.*

**Neurasthenia.**

The fundamental origin of neurasthenia must be sought for in psychical and not in physical causes. The most violent physical disturbances are, as a rule, impotent to arouse by themselves the profuse symptom complex of neurasthenia. We shall find that each patient has been the subject of antecedent circumstances which only required the collision or the illness to determine the onset of neurasthenia. If no predisposition of this kind (worry, nervous, overstrain, etc.) can be revealed, it will probably be found that the influence of a bad heredity is responsible for some definite cerebral instability.—*Dr. J. Snowman.*

**Nocturnal Incontinence.**

An hour or two of sleep in the middle of the day has sometimes been of advantage to a patient suffering from this condition.

**Adult Epilepsy.**

The occurrence of fits indistinguishable from epilepsy in a man over 25 years of age may be taken as evidence of syphilis.—*Dr. F. W. Mott.*

**Enteroptosis.**

Constipation in women is sometimes accompanied by signs of enteroptosis, and the wearing of a wide belt has had a surprisingly beneficial effect in restoring a daily action of the bowels.

**Coal-gas Poisoning.**

Several cases have been recorded where definite dementia has occurred after poisoning by coal-gas. The effect is due to the action of the carbon monoxide, and the recovery has been slow.

**Measles.**

The period of invasion is variable, and occasionally may last a week. This must be allowed for if the appearance of the rash be taken as the fixed point. In the majority of cases nine or ten days elapse from the time of infection to the first symptom, and the rash appears about thirteen to fifteen days after exposure. A quarantine of fourteen days is sufficient, provided the fauces be carefully examined on the last day.—*Dr. C. B. Ker.*

**Pseudo-General Paralysis.**

Whereas the evolution of the true general paralytic is slow, insidious, and progressive towards marasmus and death, the victim of diffuse syphilitic brain disease may, if treated, remain stationary or even improve. A most important point in the diagnosis is the fact that the pseudo-general paralytic still possesses the faculty of autocriticism and is more or less conscious of his mental deficiency. His dementia is partial and unequal rather than a diminution of intelligence as a whole. The characteristics of the individual are not so profoundly altered, nor is orientation of time and space destroyed.—*Dr. F. W. Mott.*

